# Editorial commentary on the special issue of cardiovascular diseases

**DOI:** 10.7555/JBR.37.20230800

**Published:** 2023-07

**Authors:** Editorial Board

Cardiovascular disease (CVD) is the most common cause of death worldwide, which includes a group of disorders affecting the heart and blood vessels, leading to an increasingly heavy burden on the society. Although some mounting efforts have been made to explore epidemiology, pathology and pathogenesis, and risk assessment and prevention of CVD, our understanding, treatment and prevention of the complex CVD remain the tip of the iceberg and need further investigations.

In this special issue, we have selected five different articles that are presenting recent advances in the study of CVD, particularly in myocardial function and coronary microvessels, involving sepsis-induced myocardial dysfunction, myocardial ischemia and reperfusion, and coronary microvascular obstruction.

Zhu *et al* explored the mechanism of sepsis-induced myocardial dysfunction, and demonstrated that the knockdown of 11β-HSD1 alleviated endotoxemia-induced myocardial mitochondrial injury, oxidative stress, and inflammation through the AMPK/SIRT1/PGC- 1α pathway^[1]^.

Zhou *et al *established an efficient myocardial ischemia/reperfusion (I/R) model in pigs through the median thoracic incision with a high success rate and homogeneity of the MI area, which provides a reference and a guiding significance for I/R preclinical research^[2]^.

Naryzhnaya *et al* summarized antiarrhythmic, cardioprotective, and vasoprotective effects of chronic hypoxia on enhancing cardiac tolerance to I/R, which involves the complex cellular and molecular mechanisms, such as reactive oxygen species, hormones and humoral factors, kinases, K_ATP_ channels and mitochondria, providing a basis for the new therapeutic approaches in I/R^[3]^.

Maslov and colleagues focused on the debate of the protective or negative role of ROS in I/R of the heart. They reviewed myocardial origin, production and roles of ROS, and antioxidants in clinical cardiological practice as well as pointed out that future studies using low concentrations of the selective free-radical scavengers with simultaneous detection of ROS production and cardiac injury in the isolated heart would be the key to solve the issue^[4]^.

Besides, in another review article, they concentrated on the latest research progress of coronary microvascular obstruction and summarized the triggers, contributing factors and potential pathogenesis of microvascular obstruction from both experimental and clinical data, including endothelial cell injury, microembolization and microthrombi, platelet aggregation, reactive oxygen species and Ca^2+^ overload; they also discussed current clinical treatment of MVO to map the MVO from different perspectives for future investigations^[5]^.

We hope the readers find this special issue interesting and intriguing.
